# A novel tRNA-derived fragment tRF-3022b modulates cell apoptosis and M2 macrophage polarization via binding to cytokines in colorectal cancer

**DOI:** 10.1186/s13045-022-01388-z

**Published:** 2022-12-16

**Authors:** Sicheng Lu, Xiaoman Wei, Lihuiping Tao, Dan Dong, Wenlong Hu, Qinchang Zhang, Yuquan Tao, Chengtao Yu, Dongdong Sun, Haibo Cheng

**Affiliations:** 1 The First Clinical Medical College, Nanjing University of Chinese Medicine, Nanjing, China; 2 Department of Oncology, Affiliated Hospital of Nanjing University of Chinese Medicine, Nanjing, China; 3 Jiangsu Collaborative Innovation Center of Traditional Chinese Medicine Prevention and Treatment of Tumor, Nanjing, China; 4 School of Integrated Chinese and Western Medicine, Nanjing University of Chinese Medicine, Nanjing, China

**Keywords:** tRNA-derived fragments, tRF-3022b, LGALS1, MIF, Colorectal cancer

## Abstract

tRNA-derived fragments (tRFs) are a class of small RNAs that occur when tRNAs are broken down by enzymes due to stress. Increasing reports have shown that tRFs are associated with multiple physiological and pathological processes, especially in cancers; however, very little is known of the effects and mechanisms of tRFs. Therefore, further investigation on the biological roles and clinical value of tRFs is required. In this study, we utilized whole-transcriptome sequencing to profile tRFs expression in the tissues and plasma exosomes of patients with colorectal cancer (CRC). Three tRFs (tRF-3022b, tRF-3030b and tRF-5008b) showed an increasing trend in CRC tissues compared to adjacent normal tissues. They also tended to be elevated in plasma exosomes of CRC patients compared to healthy controls. These results indicated that they may be upregulated in cancer cells and then secreted by exosomes. The knockdown of tRF-regulated factors such as AlkB homolog 3 (ALKBH3), tRNA aspartic acid methyltransferase 1 (DNMT2), angiogenin (ANG), and argonaute RISC catalytic component 2 (AGO2) could affect the expression of tRFs. Notably, we found that the decrease in the three tRFs arrests the progression of the CRC cell cycle and induces cell apoptosis. Silencing tRF-3022b could facilitate M2 macrophage polarization. Mechanistically, we found that tRF-3022b binds to galectin 1 (LGALS1) and macrophage migration inhibitory factor (MIF) in CRC cells and reduces polarization by regulating MIF in M2 macrophages. In conclusion, our study revealed the expression pattern of tRFs in both tissue and plasma exosomes and identified a novel tRF, tRF-3022b, which may affect CRC tumor growth and M2 macrophage polarization by binding to LGALS1 and MIF.

**Supplementary Information:**

The online version contains supplementary material available at 10.1186/s13045-022-01388-z.

To the Editor,

Colorectal cancer (CRC) is a heterogeneous disease and is the second and third most common cancer in females and males, respectively [1]. In recent years, an increasing number of studies have shown that, when catalyzed by enzymes, tRNA can be cleaved into small molecular fragments called tRNA-derived fragments (tRFs), which participate in the central dogma independently as amino acid carriers. Evidence has shown that tRFs exert function by directly binding to proteins [2–4].

The workflow of this study is summarized in Fig. 1a, with more details provided in Additional file 2: Fig. S1. In this study, tissues were collected from 10 patients with CRC to transcriptome sequencing and small RNA sequencing, and plasma exosomes were obtained from 10 CRC patients and 10 healthy controls to small RNA sequencing (Additional file 3: Tables S1-1, S1-2). First, we analyzed mRNA expression in CRC tissues versus matching adjacent normal tissues and carried out Gene set enrichment analysis (GSEA). The results suggested that CRC tissues were enriched with signature pathways such as cytokine-cytokine receptor interaction, IL-17 signaling pathway, NOD-like receptor signaling pathway, and ribosome biogenesis in eukaryotes (Fig. 1b, c and Additional file 4: Tables S7, 8). Meanwhile, CRC tissues and plasma exosomes demonstrated distinct patterns of tRFs expressions (Fig. 1d, e and Additional file 4: Tables S9, 10). Some studies have reported that tRFs can trigger mRNA degradation or translational repression, similar to miRNAs [5]. Therefore, we used the miRanda algorithm to identify putative target mRNAs using the KEGG database. Multiple pathways, such as the PI3K-Akt, calcium, and Rap1 signaling pathways (Additional file 2: Fig. S2a, b) were observed. Next, we regrouped the tissue samples only based on the score of specific tRFs (tissues’ tRF or plasma exosomes’ tRF) and divided the samples into high tRF_group and low tRF_group (Additional file 5: Table S21-1, S21-2). Enrichment assays showed that IL-17 signaling pathway and ribosome biogenesis in eukaryotes were also associated with the high tRF_group and low tRF_group (Fig. 1f–i and Additional file 4: Tables S11–S14). We further found that tRF-3 was the most abundant tRF in tissues (50.70%) and plasma exosomes (46.84%) (Fig. 1j, k). The tRFs derived from tRNA-Gly were relatively high in exosomes and tissues (Additional file 2: Fig. S2c, d). Three tRFs (tRF-3022b, tRF-3030b, and tRF-5008b) had relatively higher expression levels than the controls (Additional file 2: Fig. S2e), and correlation analysis suggested that some plasma exosome tRF might be from tumor tissues (Fig. 1l). We then validated three tRFs were frequently upregulated in CRC tissues and plasma exosomes compared to their normal counterparts (Additional file 2: Fig. S3a–e and Additional file 3: Tables S2, S3). We next investigated the area under the receiver-operating characteristic ROC curve (AUC) values for each biomarker (Additional file 2: Fig. S4a–e). Altogether, these results suggest that these tRFs have potential diagnostic capabilities for patients with CRC. A previous study reported that tRFs are produced from pre-tRNAs or mature tRNAs via non-random cleavage events mediated by certain proteins and enzymes [6]. Likewise, it suggested that ALKBH3, DNMT2, AGO2 and ANG may be related to the biogenesis of tRFs in CRC (Additional file 2: Fig. S5a–d).

**Fig. 1:**
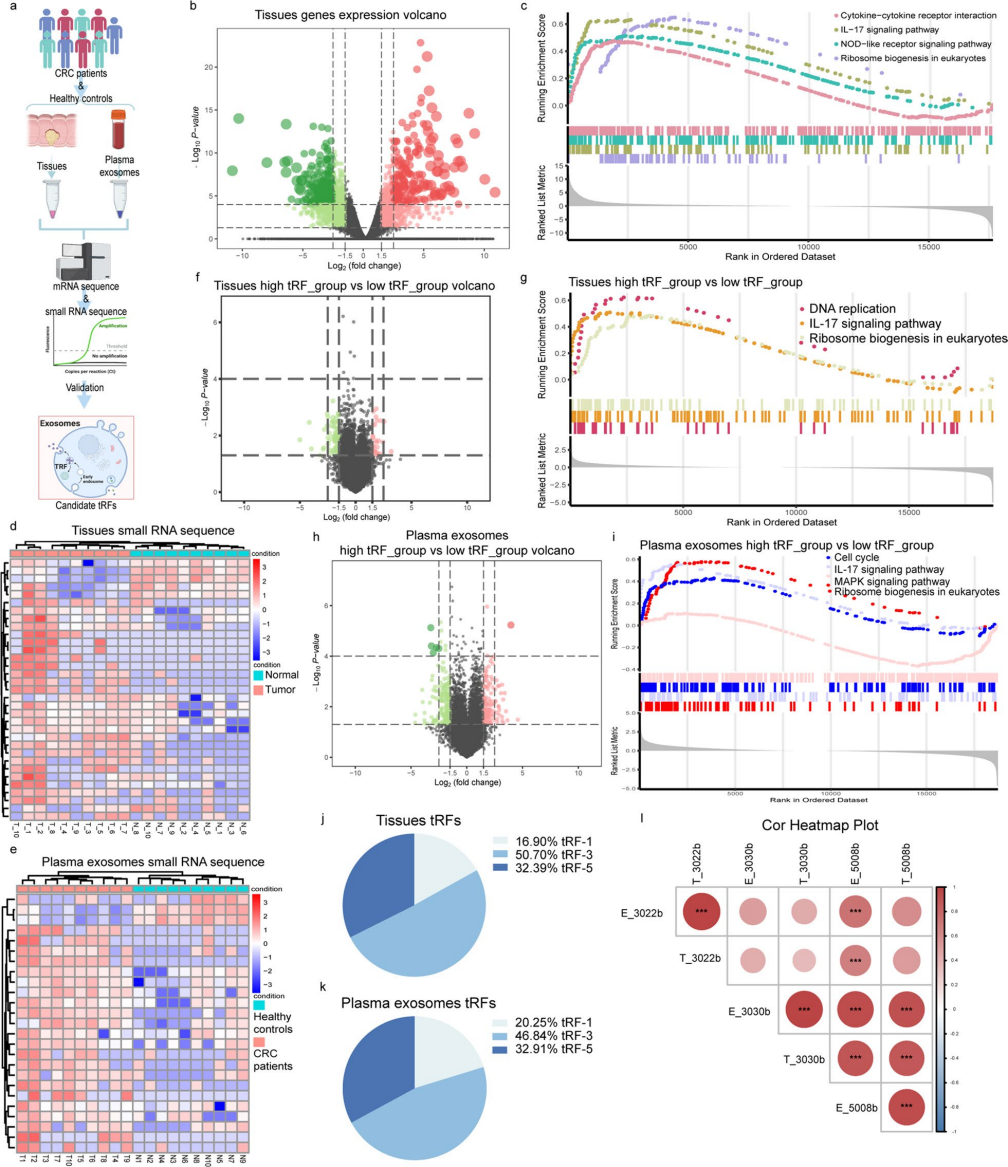
Screening and profiling of tRFs in the tissues and plasma exosomes from CRC patients and healthy controls. **a** Schematic presenting the experimental procedure of our study. **b** Volcano plot showing the gene expression profile of CRC tissues and matching adjacent normal tissues. Down-regulated genes are indicated in green; upregulated genes are indicated in red. **c** Gene set enrichment analysis of the differential gene in tissues. **d** A heatmap showing 33 differentially expressed tRFs in 10 paired CRC tissues and matching adjacent normal tissues. **e** A heatmap showing 25 differentially expressed tRFs between 10 plasma exosomes of CRC and healthy controls. **f** Volcano plot showing significant DEGs in tissues between the high tRF_group and low tRF_group. Down-regulated genes are indicated in green; upregulated genes are indicated in red. **g** GSEA plots for high tRF_group and low tRF_group in tissues. **h** Volcano plot showing significant DEGs in plasma exosomes between the high tRF_group and low tRF_group. Down-regulated genes are indicated in green; upregulated genes are indicated in red. **i** GSEA plots for high tRF_group and low tRF_group in plasma exosomes. **j** Percentage of each type of tRFs in tissues. **k** Percentage of each type of tRFs in plasma exosomes. **l** Correlation analysis of tRF-3022b, tRF-3030b and tRF-5008b between tissues and plasma exosomes. (***P < 0.001)

To investigate the specific functions of tRFs, we performed flow cytometry assay to demonstrate that knockdown of tRF-3022b, tRF-3030b, and tRF-5008b arrested the G2/M phases and promoted cellular apoptosis (Additional file 2: Figs. S6a, b and S7a, b). Subcutaneous injection of LNA_3022b-transfected HCT116 cells into nude mice attenuated tumor growth in vivo (Fig. 2a–c). Considering the presence of tRFs in plasma exosomes, we analyzed expression level of tRFs in cell-derived exosomes of various cell lines (Fig. 2d, e and Additional file 2: Fig. S8a–d). Next, we established a co-culture system and illustrated knockdown of tRF-3022b became much easier to promote M2 macrophage polarization (Additional file 2: Fig. S9a–g). Consistent with this result, qRT-PCR and flow cytometry results showed that tRF-3022b effectively modulates M2 macrophage polarization in vitro (Fig. 2f–h and Additional file 2: Fig. S9h).

**Fig. 2:**
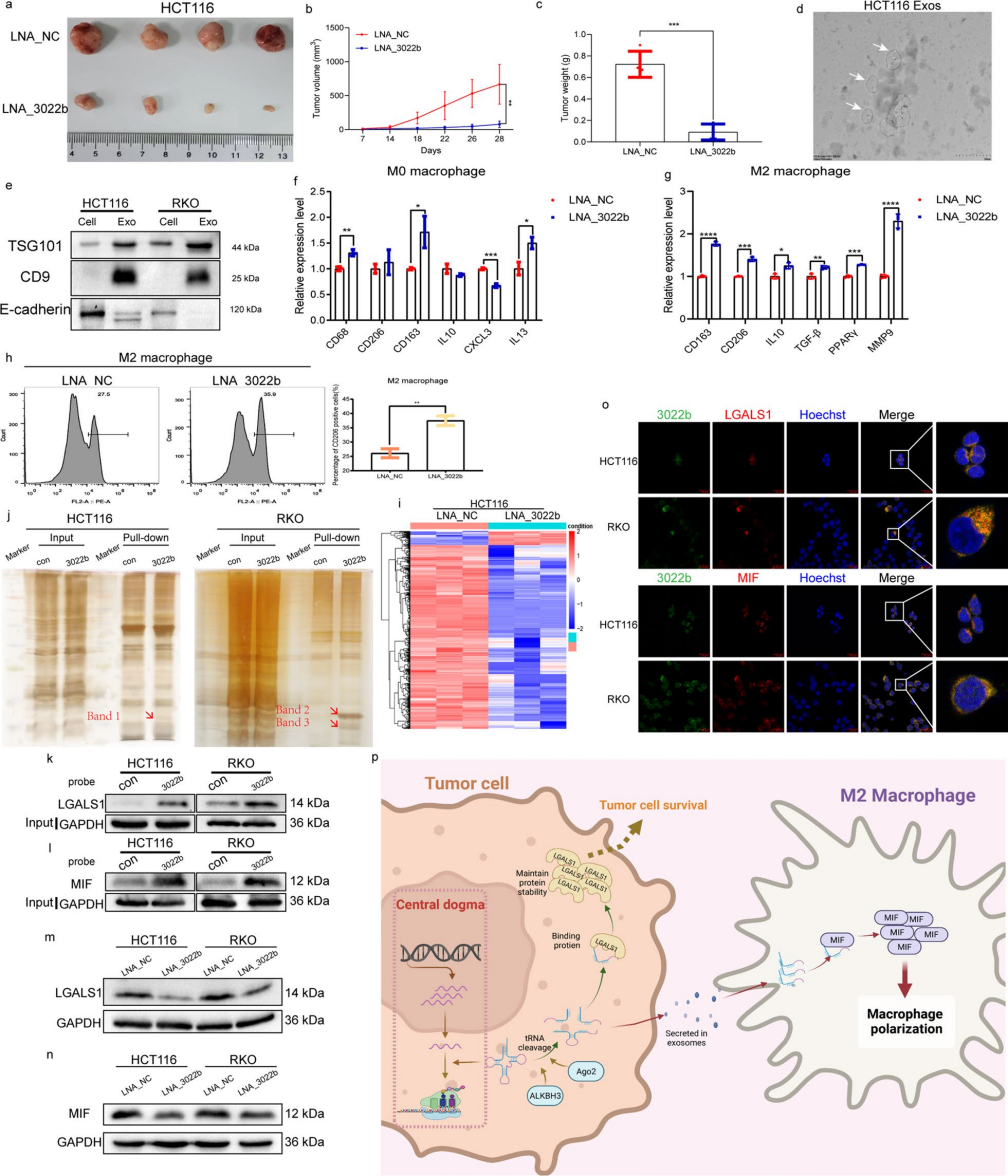
Suppression of tRF-3022b inhibits subcutaneous tumor growth and induces M2 polarization via LGALS1 and MIF. **a** Representative images of subcutaneous xenograft tumors (*n* = 4 mice per group). **b** Tumor volume at indicated time points was calculated and plotted (*n* = 4 mice per group). **c** Final weight of subcutaneous xenograft tumors was measured and quantified (*n* = 4 mice per group). **d** Transmission electron micrograph (TEM) of exosomes isolated from HCT116 cell (scale bar = 200 nm). **e** Western blot analysis of TSG101, CD9, and E-cadherin (negative control) in exosomes. Cellular lysates were used as positive loading controls. **f** mRNA levels of various cytokines and markers in the M0 macrophages were measured by qRT-PCR analysis. **g** mRNA levels of M2 macrophage markers were measured by qRT-PCR. **h** Flow cytometry was used to quantify the expression of CD206, an M2 macrophage marker. **i** Cluster heat map of the DEGs between tRF-3022b-knockdown and negative control HCT116 cells. **j** Silver SDS-PAGE gel image shows proteins immunoprecipitated by the tRF-3022b probe in HCT116 and RKO cells. **k**–**l** Western blot analysis of products from RNA pull-down assays using the tRF-3022b probe and a control probe suggested LGALS1 and MIF in HCT116 and RKO cells. **m** Protein levels of LGALS1 in CRC cells with tRF-3022b knockdown. **n** Protein levels of MIF in CRC cells with tRF-3022b knockdown. **o** Immunofluorescence images of tRF-3022b with LGALS1 and MIF co-staining. Cell nuclei were counterstained with Hoechst. Scale bar: 20 μm. **p** Diagram outlining discussed hypotheses about the roles of tRF-3022b in the CRC tumor microenvironment. (*P < 0.05, **P < 0.01, ***P < 0.001, ****P < 0.0001)

Next, RNA sequencing analysis identified the target genes regulated by tRF-3022b and bioinformatics analysis (KEGG and IPA analysis) demonstrated they were involved in tight interactions with cytokine-associated tumor microenvironment. (Fig. 2i, and Additional file 2: Fig. S10a–d and Additional file 4: Tables S15–S17). Moreover, we performed RNA pull-down assay to detect tRF-3022b-associated proteins, and galectin 1 (LGALS1) and macrophage migration inhibitory factor (MIF) attracted our attention (Fig. 2j and Additional file 4: Tables S18–S20). Then, western blot, RNA immunoprecipitation (RIP)-qPCR, RNA FISH and immunofluorescence results collectively evidenced that the association of tRF-3022b with MIF and LGALS1 (Fig. 2k–o and Additional file 2: Fig. S10e, f). Notably, western blot showed that tRF-3022b could precipitate MIF in THP-1 lysates (Additional file 2: Fig. S10g). Finally, we overexpressed LGALS1 and MIF in CRC cells and validated their oncogenic functions in CRC [7, 8] (Additional file 2: Fig. S11a–f).

In summary, we demonstrate, for the first time, tRF-3022b is specifically overexpressed in CRC and the oncogenic role in CRC progression through a regulatory pathway by binding to LGALS1 and MIF in CRC cells. In addition, tRF-3022b modulates M2 macrophage polarization by binding to MIF (Fig. 2p) (Additional file 1).

## Supplementary Information

**Additional file 1**. Materials and methods.

**Additional file 2**. Supplementary figures and legends.

**Additional file 3**. **Table S1-1**: Basic characteristics of patients who provided tissues and plasma exosomes for transcriptome sequencing and small RNA sequencing. **Table S1-2**: Basic characteristics of healthy controls who provided plasma exosomes for transcriptome sequencing and small RNA sequencing. **Table S2**: Clinical information of CRC patients who provided tissues for validation. **Table S3**: Clinical information of CRC patients who provided plasma exosomes for validation. **Table S4**: Primers and probes were used in this study. **Table S5**: Sequences of siRNAs and mimics or locked nucleic acids (LNA) were used in this study. **Table S6**: Antibodies were used in this study.

**Additional file 4**. **Table S7**: Gene expression levels in CRC tissues and matching adjacent normal tissues. **Table S8**: Gene set enrichment analysis (GSEA) of differentially expressed genes in CRC tissues and matching adjacent normal tissues. **Table S9**: List of 33 differentially expressed tRFs in CRC tissues and matching adjacent normal tissues. **Table S10**: List of 25 differentially expressed tRFs in plasma exosomes from CRC patients and HCs. **Table S11**: Gene expression levels of high tRF_group and low tRF_group in tissues. **Table S12**: Gene set enrichment analysis (GSEA) of differentially expressed genes of high tRF_group and low tRF_group in tissues. **Table S13**: Gene expression levels of high tRF_group and low tRF_group in plasma exosomes. **Table S14**: Gene set enrichment analysis (GSEA) of differentially expressed genes of high tRF_group and low tRF_group in plasma exosomes. **Table S15**: Differentially expressed genes regulated by tRF-3022b-knockdown in HCT116 cells (q-value < 0.05, fold change > 2, or fold change < 0.5). **Table S16**: KEGG analysis of DEGs in tRF-3022b-knockdown and control cells. **Table S17**: IPA pathway enrichment analysis of differentially expressed genes in tRF-3022b-knockdown and control cells. **Table S18**: Band 1 mass spectrometry results. **Table S19**: Band 2 mass spectrometry results. **Table S20**: Band 3 mass spectrometry results.

**Additional file 5**. **Table S21-1**: Grouping of high tRF_group and low tRF_group in tissues. **Table S21-2**: Grouping of high tRF_group and low tRF_group in plasma exosomes.

## Data Availability

All data generated or analyzed during this study are included in this published article and its supplementary information files.

## Abbreviations

CRC: Colorectal cancer
tRFs: TRNA-derived fragments
ALKBH3: AlkB homolog 3
DNMT2: TRNA aspartic acid methyltransferase 1
ANG: Angiogenin
AGO2: Argonaute RISC catalytic component 2
LGALS1: Galectin 1
MIF: Macrophage migration inhibitory factor
TME: Tumor microenvironment
HCs: Healthy controls
DEGs: Differentially expressed genes
GSEA: Gene set enrichment analysis
LNA: Locked nucleic acid
TEM: Transmission electron microscopy
IPA: Ingenuity pathway analysis
