# Supplementary material for: A novel tRNA-derived fragment tRF-3022b modulates cell apoptosis and M2 macrophage polarization via binding to cytokines in colorectal cancer
Source: J Hematol Oncol. 2022 Dec 16;15:176. doi: 10.1186/s13045-022-01388-z (PMC9756499; doi:10.1186/s13045-022-01388-z)
Supplement: Supplementary file 1 — Additional file 1. Materials and methods. [file 13045_2022_1388_MOESM1_ESM.docx]

**Materials and Methods**

**Clinical samples collection and processing**

Specimens were collected from patients with colorectal cancer and healthy controls (HCs) in the Jiangsu Province Hospital of Chinese Medicine between October 2020 and May 2021. The collected blood samples were centrifuged at 3,000×g for 10 min at 4°C in a swing bucket centrifuge to harvest plasma supernatant within 4 h and then stored at -80°C until analysis. In addition, fresh tissues were collected, snap-frozen in liquid nitrogen, and stored at -80°C. 10 matched samples of the CRC tissues and corresponding adjacent normal tissues (defined as 5 cm~8 cm from the lesion) were collected for transcriptomic sequencing and small RNA sequencing. Due to the contamination of transcriptome data in one case, 9 paired patient samples were finally selected for combined analysis of transcriptome results and small RNA sequencing results. Meanwhile, the 10 CRC patients and 10 HCs’ blood samples were used for exosomal small RNA sequencing (Table S1-1, S1-2). Paired tumor and adjacent non-neoplastic tissues from 51 CRC patients without chemotherapy, radiation, or any other kinds of treatment were obtained for validation (Table S2). Similarly, blood samples from 19 CRC patients (Table S3) and 20 HCs were collected using standard procedures and stored at -80°C until analysis.

This study was reviewed and approved by the Affiliated Hospital of Nanjing University of Chinese Medicine (Nanjing, China; ID: 2020NL-09404). The CRC stage was classified according to the seventh edition of TNM staging criteria issued by the American Joint Committee on Cancer Staging Manual. All samples have been collected and utilized following the Declaration of Helsinki, and written informed consent was obtained from the patients.

**Exosome isolation**

For plasma exosomes, the blood samples were processed as described above^[1]^. Exosomes were isolated using an RiboTM Exosome Isolation Reagent (a precipitation reagent for plasma or serum, C10110-2, Ribobio, China) following the manufacturer’s instructions. Briefly, plasma samples were centrifuged at 2000×g for 20 min at 4°C to remove possible residual cell debris after thawing the stored samples. Clarified plasma samples were then transferred to a clean tube containing one-third volume of Ribo Exosome Isolation Reagent. The mixtures were incubated overnight at 4°C and then centrifuged at 15,000×g for 2 min. The resulting exosomes were slightly washed and re-suspended in TRIzol reagent (Invitrogen, CA, USA).

For cell-derived exosomes, cells were cultured in medium with FBS which was adopted to ultracentrifugation at 110,000xg at 4°C for 48 h, then the cell culture medium was harvested and centrifuged at 300xg for 10 min at 4°C. Then the obtained supernatant was centrifuged at 2000xg for 10 min, 10,000xg for 30 min and 110,000xg for 70 min sequentially (all steps were performed at 4°C). The isolated exosomes were prepared for the following experiment.

**Small RNA library sequencing and analysis**

Briefly, total RNA from tissues of 10 CRC patients and plasma exosomes of 10 CRC patients and 10 HCs were extracted with TRIzol reagent. Sequencing libraries were constructed with purified and integrated small RNA using the TruSeq Small RNA Sample Prep Kits (Illumina Cat#: RS-200-0012) according to the manufacturer’s protocol. The size distribution and quantity of sequencing libraries were determined using Bioanalyzer 2100 DNA-HS Kit (Agilent Cat#: 5067-4626) on Agilent Technologies 2100 Bioanalyze.

The raw data was cleaned through removing the adapter sequences and low-quality reads were filtered out. Further, these reads ranging from 15-30 nt insertion were aligned and subjected to tRFdb databases (http://genome.bioch.virginia.edu/trfdb/search.php)^[2]^. Then the reads matched to the databases were counted and classified into tRF-1, tRF-3 and tRF-5. Differential expression between tRFs was assessed by DEseq algorithm. We defined the statistical criteria for selecting aberrant-expressed tRFs using a q < 0.05 with |log2FoldChange|>1.

**Transcriptomic sequencing**

Total RNA was extracted using the TRIzol reagent according to the manufacturer’s protocol and was sent to OE Biotech Co., Ltd. (Shanghai, China). Then the libraries were constructed using TruSeq Stranded mRNA LT Sample Prep Kit (Illumina, San Diego, CA, USA) according to the manufacturer’s instructions. The quantified and validated libraries were sequenced on the Illumina HiSeq X Ten platform and 150 bp paired-end reads were generated. The differentially expressed genes (DEGs) were identified using the DESeq (2012) R package, and these DEGs were undergone Gene ontology and KEGG pathway analysis respectively.

**Quantitative real-time PCR**

Total RNA was isolated using the TRIzol reagent according to the manufacturer’s instruction. The quality and quantity of RNA were measured with an NanoDrop 2000 spectrophotometer (Thermo Fisher). Reverse transcription was performed using Hifair® Ⅱ 1st Strand cDNA Synthesis Kit (gDNA digester plus) to cDNA (Yeasen, Shanghai) or Mir-X™ miRNA First Strand synthesis kit (Takara) for RNA and tRFs according to the protocols respectively. Quantitative expression was conducted on the StepOne Plus real-time PCR system (Applied Biosystems, CA, USA) with Hieff® qPCR SYBR Green Master Mix (High Rox Plus) (Yeasen, Shanghai). The primer sequences were shown in Table S4. Meanwhile, we used β-actin and U6 as the internal references for mRNAs and tRFs, respectively.

**Plasmids**

All siRNAs targeting ALKBH3, DNMT2, ANG and AGO2 were purchased from Tsingke Biotech and were performed according to the manufacturer’s instructions, the sequences were listed in Table S5. The full length cDNAs of LGALS1 (Gene ID: NM_002305.4) and MIF (Gene ID: NM_002415.2) were synthesized by Tsingke Biotech, and sub-cloned into plv vector.

**tRFs *in vitro* modulation**

The tRF mimics and tRF antisense locked nucleic acid (LNA) oligonucleotides were synthesized by Tsingke Biotech, the sequences were shown in Table S5.

**Transmission electron microscopy**

The isolated exosome was suspended in approximately 20 μl PBS buffer and dropped onto a carbon-coated copper grid (200 mesh), allowed to dry for 5 min at room temperature. The sample was then stained with 30 μl uranyl acetate for 3 min and the residual reagent was removed with filter paper and air dried for 30 min. The copper mesh was observed and photographed under a transmission electron microscope (TEM, HT7800; Hitachi, Japan).

**Cell culture**

The human colorectal cancer cell lines HCT116, HCT8, HT29 and RKO, the fetal human colon cell line FHC and the human leukemia monocyte cell line THP-1 were purchased from the Shanghai Cell Bank, Type Culture Collection Committee of Chinese Academy of Science (Shanghai, China). The cells were routinely maintained in RPMI1640 (Basalmedia Technologies, Shanghai) supplemented with 10% FBS (Sijiqing, Hangzhou) and 1% penicillin/streptomycin (Basalmedia Technologies, Shanghai) at 37°C in a humidified 5% CO_2_ incubator.

The cell co-culture system consists of a transwell system with a porous membrane filter (0.4 μm pore size; Labselect) and 6-well plastic culture plates. THP-1 cells or M0 macrophages were plated on the upper inserts, and HCT116/RKO transfected with LNA_NC and LNA_3022b cells were seeded onto the bottom of the culture plate and cocultured for 48 h.

**Cell transfection**

For siRNA, mimic and LNA transfection, cells were transfected via liposome-mediated transfection using the LipoRNAi™ Transfection Reagent (#C0535, Beyotime, China) according to the manufacturer’s protocol. For LGALS1 and MIF overexpression, cells were transfected with plv-LGALS1 and plv-MIF plasmids using Lipo8000™ Transfection Reagent (#C0533, Beyotime, China) according to the manufacturer’s instructions.

***In vivo* tumorigenicity**

HCT116 cells transfected with LNA_NC and LNA_3022b (3×10^6^ cells in 100 μl PBS) were subcutaneously injected into the dorsal flank of 4-week-old female BALB/c nude mice (5 mice/group). The control groups in which mice were subcutaneously injected by HCT116 cells transfected with LNA_NC. The Tumor volume was monitored after inoculation according to the following formula: volume = (tumor length)^2^× (tumor width) ×0.5. All experimental procedures were approved by the Animal Ethics Committee of the Nanjing University of Chinese Medicine.

**M0 macrophages and polarization of M2 macrophages**

The THP-1 cells were differentiated into M0 macrophages by incubating in 200 ng/ml Phorbol 12-myristate 13-acetate (PMA) for 48 h, briefly. For M2-polarized macrophages, THP-1 cells were treated with 200ng/ml PMA for 24 h and then cultured with 25 ng/ml IL-4 and 25 ng/ml IL-13 for 48 h.

**Western blot analysis**

Total proteins of tissues and cells were extracted by RIPA buffer (Beyotime) supplemented with InStab™ Protease Inhibitor Cocktail (Yeasen) according to its protocol. Following denatured at 100°C for 10 min, equal amount of total protein (20 μg) was loaded on SDS-PAGE gel and transferred to PVDF membranes (Millipore, MA, USA). After blocking the non-specific binding sites with Protein Free Rapid Blocking Buffer (EpiZyme), the membranes were washed with TBST and probed with primary antibodies at 4°C overnight. The next day, the protein was washed with TBST three times and then incubated with secondary antibodies at room temperature. GAPDH was used as an internal reference. Antibodies were listed in Table S6.

**Flow cytometry analysis**

For cell cycle analysis, HCT116 and RKO cells transfected with LNA_NC, LNA_3022b, LNA_3030b and LNA_5008b were harvested, washed and fixed in ice-cold 70% ethanol for overnight, then resuspended in 250 μl PBS with 5 μl RNase A (10 mg/ml) and incubated for 1 h. After that, the samples were added 10 μl PI solution (10 μg/ml) incubated in the dark at room temperature for 10 min, they were analyzed by CytoFLEX flow cytometry (Beckman Coulter).

For cell apoptosis analysis, HCT116 and RKO cells transfected with LNA_NC, LNA_3022b, LNA_3030b and LNA_5008b were detected with Alexa Fluor 647 Annexin V/PI Apoptosis Detection Kit (Yeasen). Briefly, cells were washed in cold PBS two times and collected with 0.25% trypsin (EDTA-free) in culture medium. Later, the supernatant was removed after centrifugation at 600 g for 3 min. Then, cells were diluted to 10^6^ cells/ml in binding buffer. 100 µl of the solution was transferred to a 1.5 ml tube followed by the addition of 5 µl of Annexin V-Alexa Fluor 647 and 10 µl of PI to every sample and incubated at room temperature for 15 min. The results were analyzed by CytoFLEX flow cytometry within 1 h.

For macrophage immunophenotyping, THP-1 cells co-cultured with HCT116/RKO transfected with control, 3022b, LNA_NC and LNA_3022b were harvested and washed in PBS. Macrophages can be differentiated into classically activated (M1) and alternatively activated (M2) macrophages. These macrophages showed stereotypic in cell surface marker expression. In details, CD68 is a well-characterized macrophage (M0 macrophage) marker in humans ^[3]^. CD163 and CD206 are commonly used in the identification and screening of M2 macrophages, especially CD206 ^[4,5]^. Because of this, we assessed the levels of CD68, CD163, and CD206 to judge the situation of macrophage differentiation. For CD68 staining, THP-1 cells were permeabilized and fixed with Fixation/Permeabilization buffer (eBioscience) for 60 min, then cells were stained with anti-CD68-FITC at 4°C for 30 min. For CD206 staining, M2 macrophage cells were stained with anti-CD206-PE (eBioscience) at 4°C for 30 min directly.

**Biotin-labeled RNA pull down assay**

Tsingke Biotech synthesized the biotin labelled tRF probes and control probes (Table S5). Biotin-labeled RNA pull-down was conducted according to the manufacturer’s instructions of the Pierce™ Magnetic RNA-Protein Pull-Down Kit (Thermo). Briefly, Cells lysates prepared in the IP lysis buffer (Beyotime) were incubated with biotin-coupled probe of tRFs which was pre-bound on magnetic beads. After overnight incubation at 4°C, beads were magnetically separated and washed four times. Then, the beads were boiled in SDS buffer for western blot detection.

**Silver staining and mass spectrometry analysis**

After pull down assay performed as describe above, supernatant mixed with loading buffer was subjected to SDS-page electrophoresis. Next, we stained the gels according to the manufacturer's instructions of Protein Silver Stain Kit (Beyotime, Shanghai). Stained gel bands were retrieved and sent to Shanghai Luming Biotechnology CO., Ltd. for LC-MS/MS analysis.

**RNA FISH and** **immunofluorescence**

Fluorescent in situ hybridization (FISH) experiments were performed to detect tRF-3022b in CRC cells using the Ribo™ Fluorescent In Situ Hybridization Kit (RiboBio) according to the protocol. FAM-labelled tRF-3022b probes (Table S5) were synthesized by TsingKe. Primary antibodies of LGALS1 (1:200, CST), MIF (1:200, CST) and secondary antibody (1:500, A0516, Beyotime) were used for immunofluorescence staining. Cell nuclei were counterstained with Hoechst. Images were acquired by Zeiss LSM-710 confocal microscope (Leica).

**CCK-8**

The proliferation rate of cells was detected by the Cell Counting Kit-8 assay (Yeasen). About two thousand cells were seeded in a 96-well plate, and CCK-8 solution were added to each well at the same time every day and incubated for another 2 h at 37°C, the absorbance at 450 nm of the experimental wells was measured with an automatic microplate reader (SPARK 10M, TECAN).

**RNA immunoprecipitation (RIP)**

RIP assay was conducted with RIP kit (P0101, Geneseed) according to vendor’s illustrations. In brief, cell lysates were cultured with Dynabeads coated with MIF (ab175189, Abcam) antibody or IgG (2729S, CST) antibody overnight at 4°C, and interested RNAs were eluted from immunoprecipitated complex and purified for further detection of enriched tRF-3022b by qRT-PCR.

**Ingenuity Pathway Analysis (IPA)**

Bioinformatic analysis of differentially expressed genes was performed using QIAGEN’s Ingenuity® Pathway Analysis which visualizes and explores molecular interactions.

**Gene Set Enrichment Analysis (GSEA)**

The GSEA enrichment analysis was performed on R version 4.1.0. The R Packages such as “tidyverse”, “clusterProfiler”, “org.Hs.eg.db”, “limma”, “GSVA”, and “openxlsx” were used in the process of GSEA analysis. The method of GSEA analysis of high tRF_group and low tRF_group was referenced in Liu’s article ^[6]^. We first calculated the scores of specific tRFs (differently expressed in CRC tissues or plasma exosomes) in each sample by the GSVA algorithm. Then, we regrouped the samples only based on the score, not considering the sample type (such as tumor or not tumor). Next, we filtered out the sample of top 30%-high scores (referenced as high tRF_group) and the bottom 30%-low scores (referenced as low tRF_group). The dichotomous value and the number of patients in each group are shown in Table S21-1 and Table S21-2. At last, GSEA analysis was performed between these two groups with mRNAs. Although these GSEA analysis could not draw precise conclusions about tRFs’ function, it gives us direction for subsequent laboratory experiments.

**Statistical analysis**

Statistical analysis was performed with IBM SPSS Statistics 5.0, GraphPad Prism version 7.0. The results were presented as the means ± S.D., and the data were subjected to Student’s t-test or Pearson’s correlation analysis. A p value of < 0.05 was considered to be statistically significant. Receiver operating characteristic (ROC) curves were plotted through CRC tissues and matching adjacent normal tissues, CRC patients’ plasma exosomes and HCs’ plasma exosomes, respectively. The area under the ROC curve (AUC) was measured. **P*<0.05, ***P*<0.01, ****P*<0.001, *****P*<0.0001; ns, no significant.

[1]Sun L, Zhu W, Zhao Pet al. Long noncoding RNA UCA1 from hypoxia-conditioned hMSC-derived exosomes: a novel molecular target for cardioprotection through miR-873-5p/XIAP axis [J]. Cell Death Dis, 2020, 11(8): 696.

[2]Kumar P, Mudunuri S B, Anaya Jet al. tRFdb: a database for transfer RNA fragments [J]. Nucleic Acids Res, 2015, 43(Database issue): D141-145.

[3]Genin M, Clement F, Fattaccioli Aet al. M1 and M2 macrophages derived from THP-1 cells differentially modulate the response of cancer cells to etoposide [J]. BMC Cancer, 2015, 15: 577.

[4]Chen Y, Zhang S, Wang Qet al. Tumor-recruited M2 macrophages promote gastric and breast cancer metastasis via M2 macrophage-secreted CHI3L1 protein [J]. J Hematol Oncol, 2017, 10(1): 36.

[5]Yang Y, Guo Z, Chen Wet al. M2 Macrophage-Derived Exosomes Promote Angiogenesis and Growth of Pancreatic Ductal Adenocarcinoma by Targeting E2F2 [J]. Mol Ther, 2021, 29(3): 1226-1238.

[6]Liu Z, Zhao Q, Zuo Z Xet al. Systematic Analysis of the Aberrances and Functional Implications of Ferroptosis in Cancer [J]. iScience, 2020, 23(7): 101302.
