# Supplementary material for: A novel tRNA-derived fragment tRF-3022b modulates cell apoptosis and M2 macrophage polarization via binding to cytokines in colorectal cancer
Source: J Hematol Oncol. 2022 Dec 16;15:176. doi: 10.1186/s13045-022-01388-z (PMC9756499; doi:10.1186/s13045-022-01388-z)
Supplement: Supplementary file 2 — Additional file 2. Supplementary figures and legends. [file 13045_2022_1388_MOESM2_ESM.pdf]

Figure S1

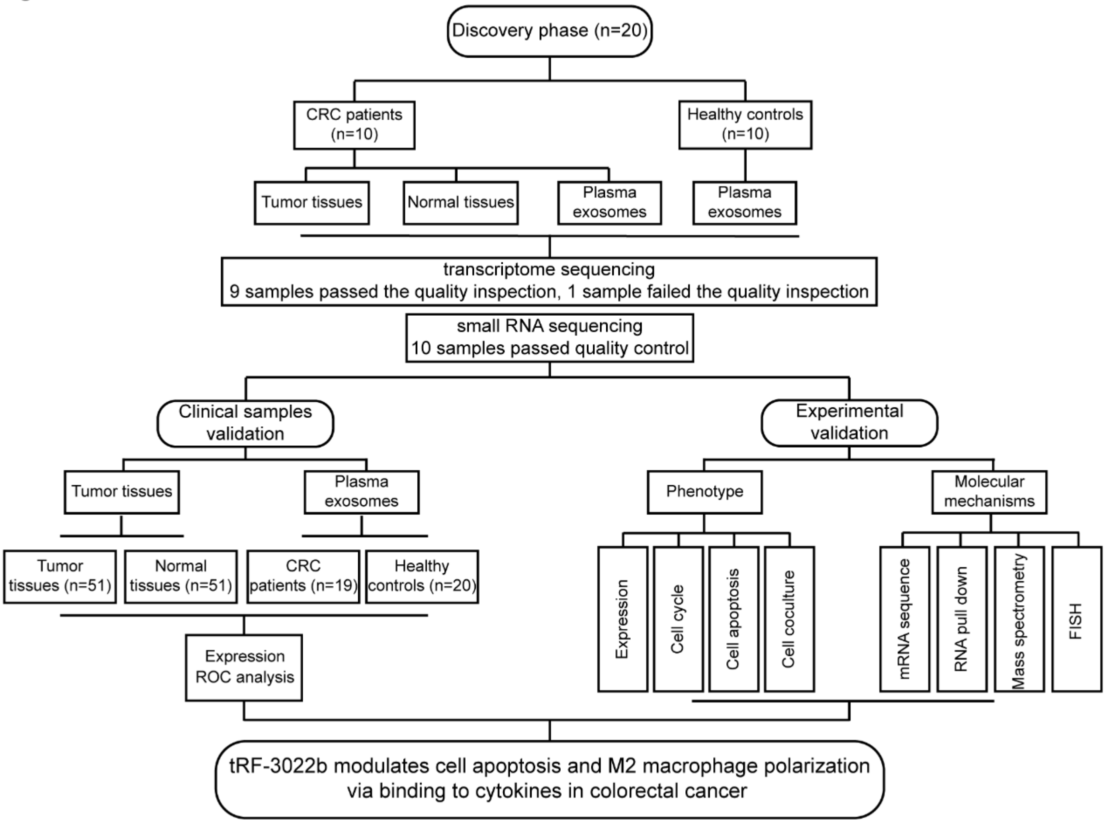

Figure S1. Flowchart of the study.

Figure S2

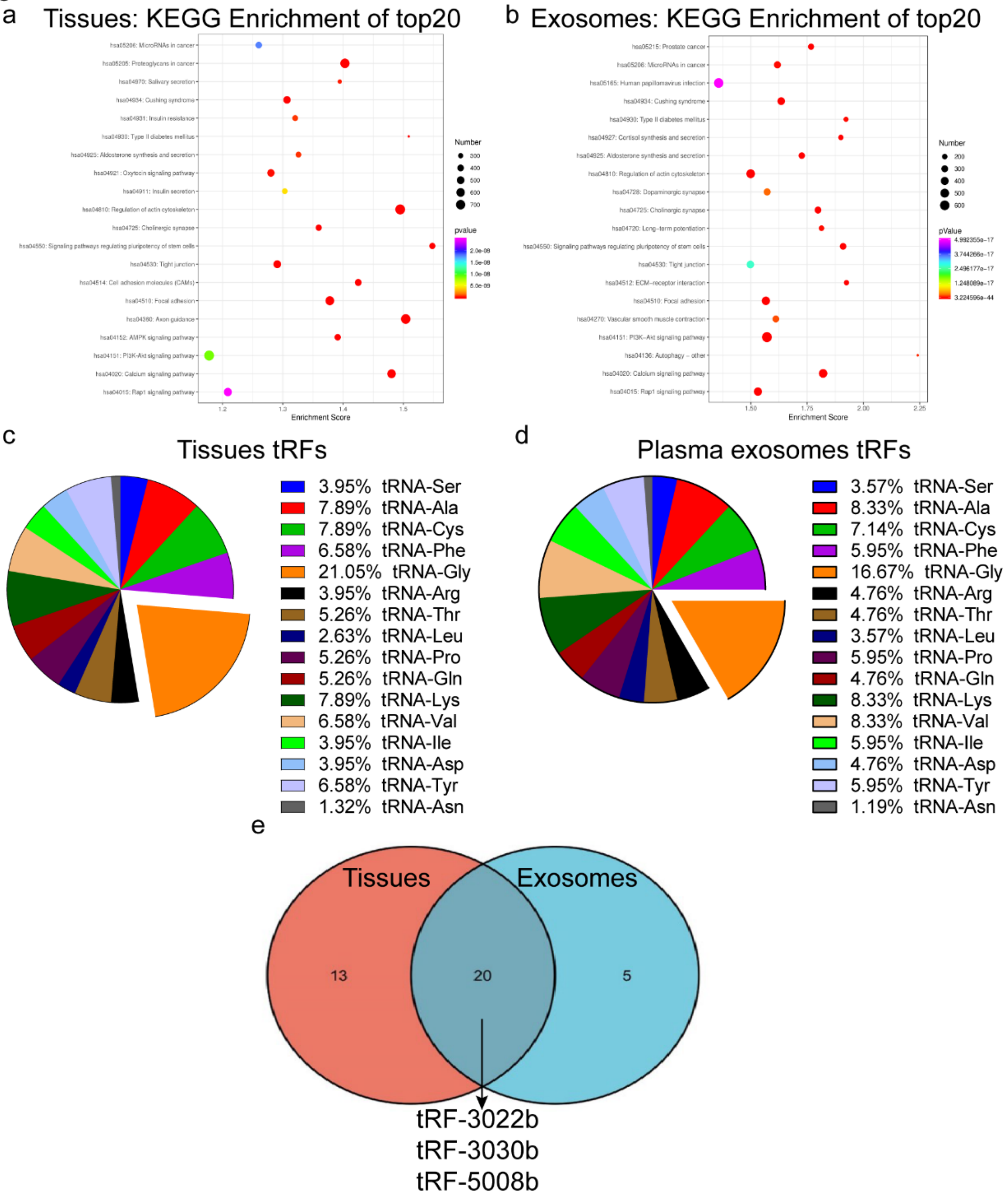

Figure S2. Functional annotation of predicted target mRNAs of differentially expressed tRFs and screening of candidate tRFs. (a-b) The top 20 signaling pathways were potentially identified by KEGG analysis of predictive target genes in tissues and plasma exosomes. (c-d) Percentage of each type of tRFs in the tissues and plasma exosomes. (e) Venn diagram showing overlap of tRFs in tissues and plasma exosomes.

Figure S3

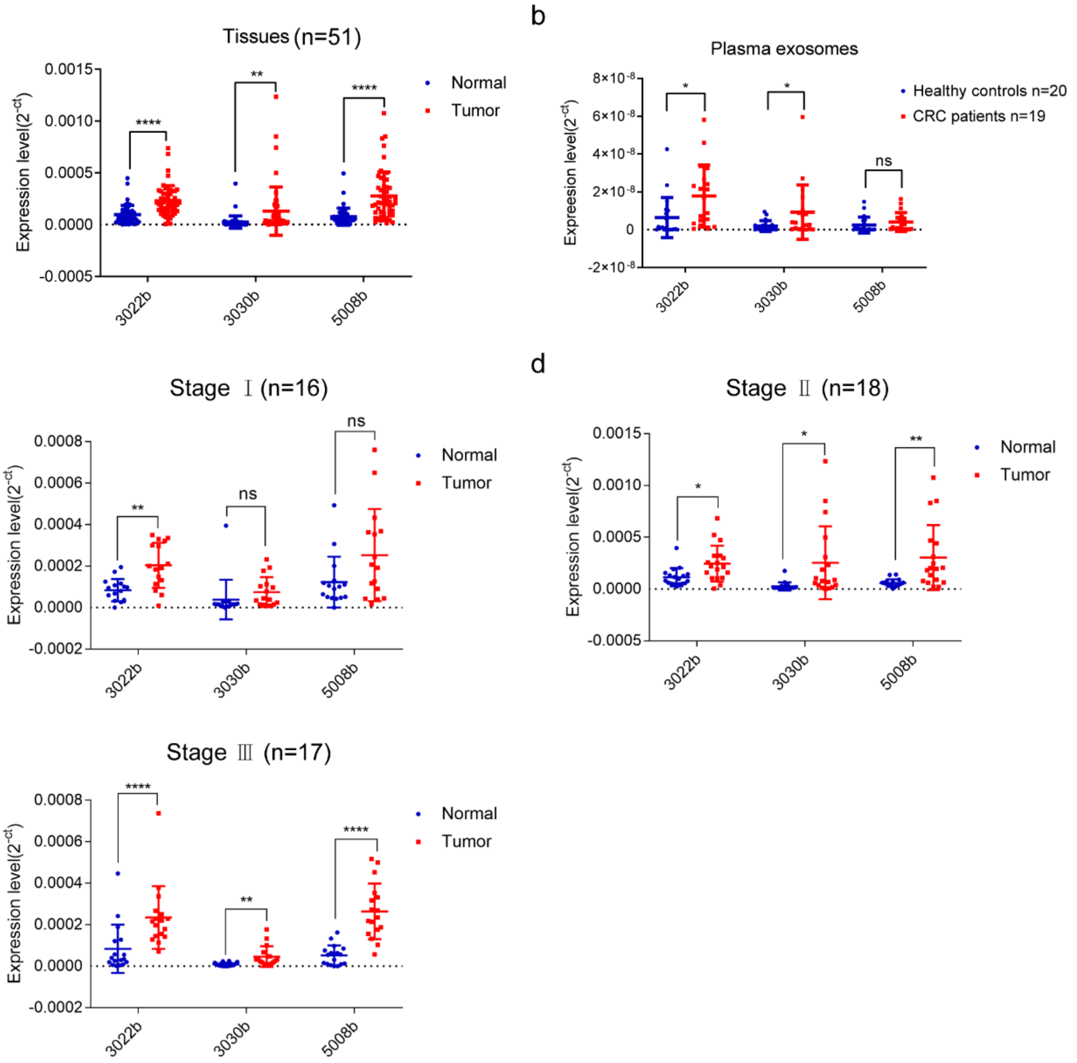

Figure S3. Levels of tRF-3022b, tRF-3030b, and tRF-5008b in tissues and plasma exosomes. (a) The expression of tRF-3022b, tRF-3030b and tRF-5008b in tissues from CRC patients (n = 51). (b) The expression of tRF-3022b, tRF-3030b and tRF-5008b in plasma exosomes from CRC patients (n = 20) and HCs (n = 19). (c-e) The amount of three tRFs in tissues of patients with different pathological stages of CRC (stage I, n = 16; stage II, n = 18; stage III, n = 17). (**P* < 0.05, ***P* < 0.01, *****P* < 0.0001, ns = not significant).

Figure S4

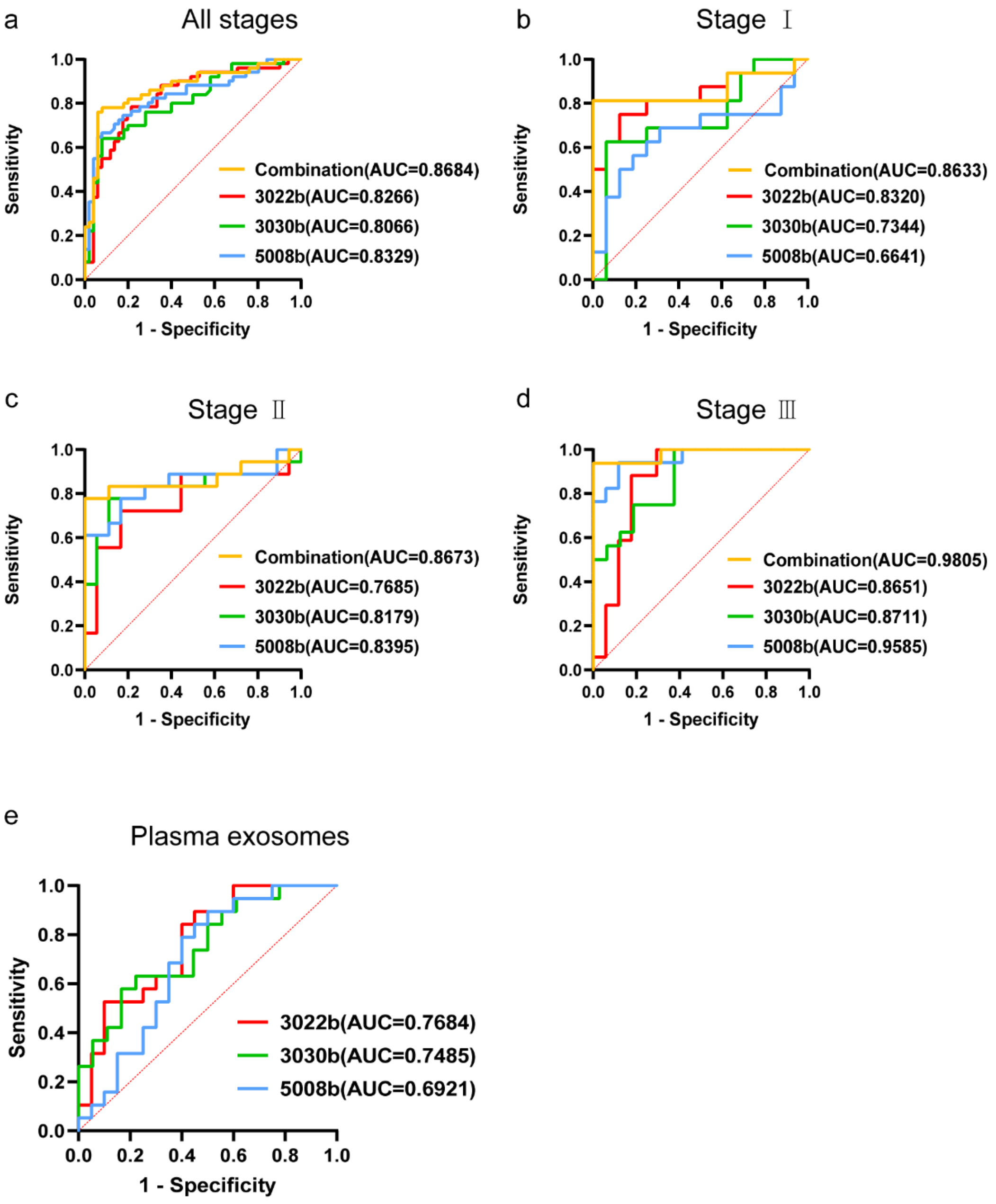

Figure S4. ROC curve analysis was used to evaluate the diagnostic values of tRF-3022b, tRF-3030b, and tRF-5008b. (a-d) ROC curves of the three tRFs in tissues and matching adjacent normal tissues from CRC patients (n = 51). (e) ROC curves of the three tRFs in plasma exosomes from patients with CRC (n = 19) and HCs (n = 20).

Figure S5

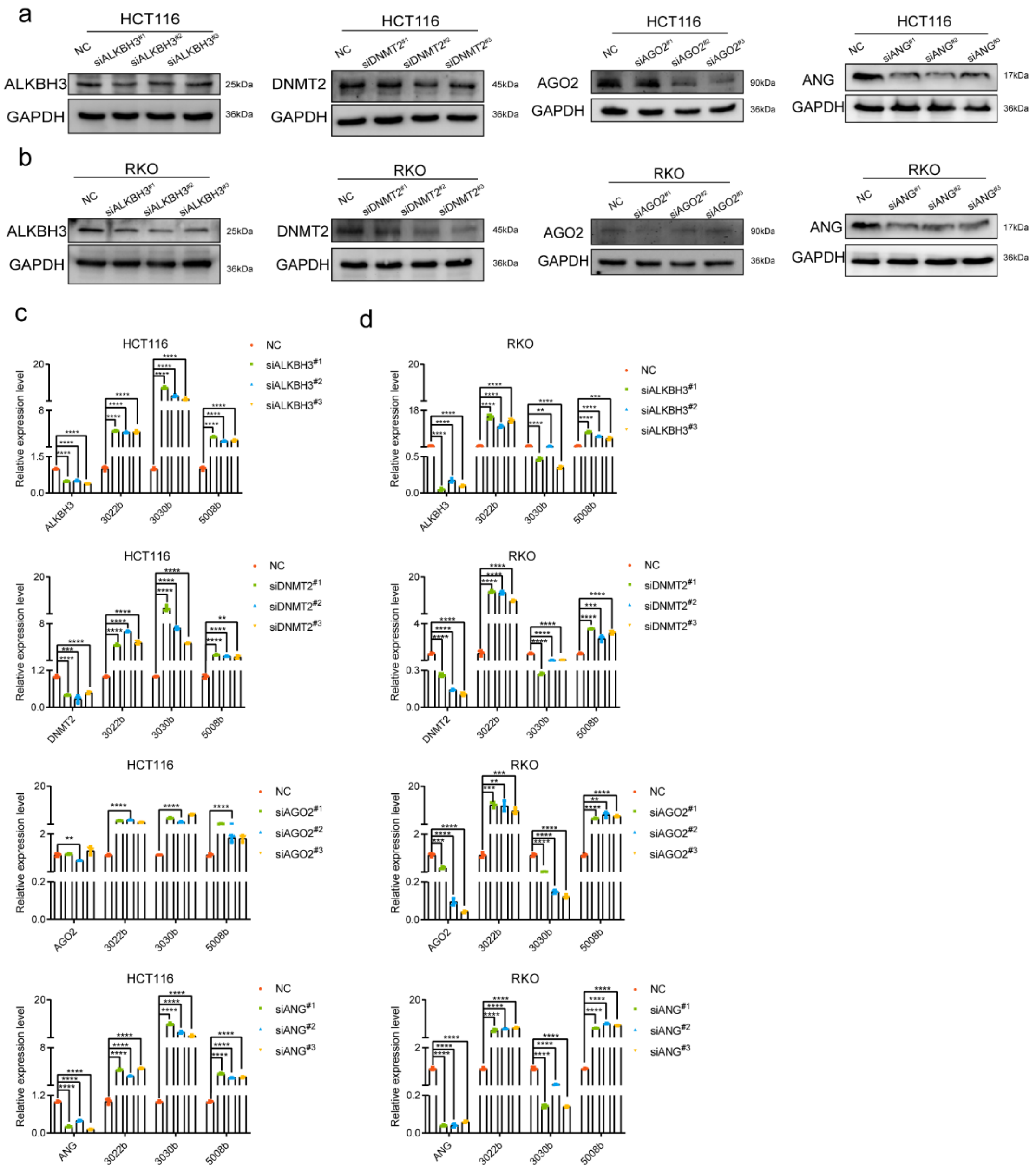

Figure S5. Enzymes are involved in the production of tRFs. (a-b) The protein expression of ALKBH3, DNMT2, ANG and AGO2 in HCT116 (a) and RKO (b) transfected with the negative control vector and targeted siRNAs for 48 h was detected by western blot. (c-d) qRT-PCR analysis of tRF-3022b, tRF-3030b and tRF-5008b in HCT116 (c) and RKO (d) transfected with the negative control vector and targeted siRNAs. (***P* < 0.01, ****P* < 0.001, *****P* < 0.0001).

Figure S6

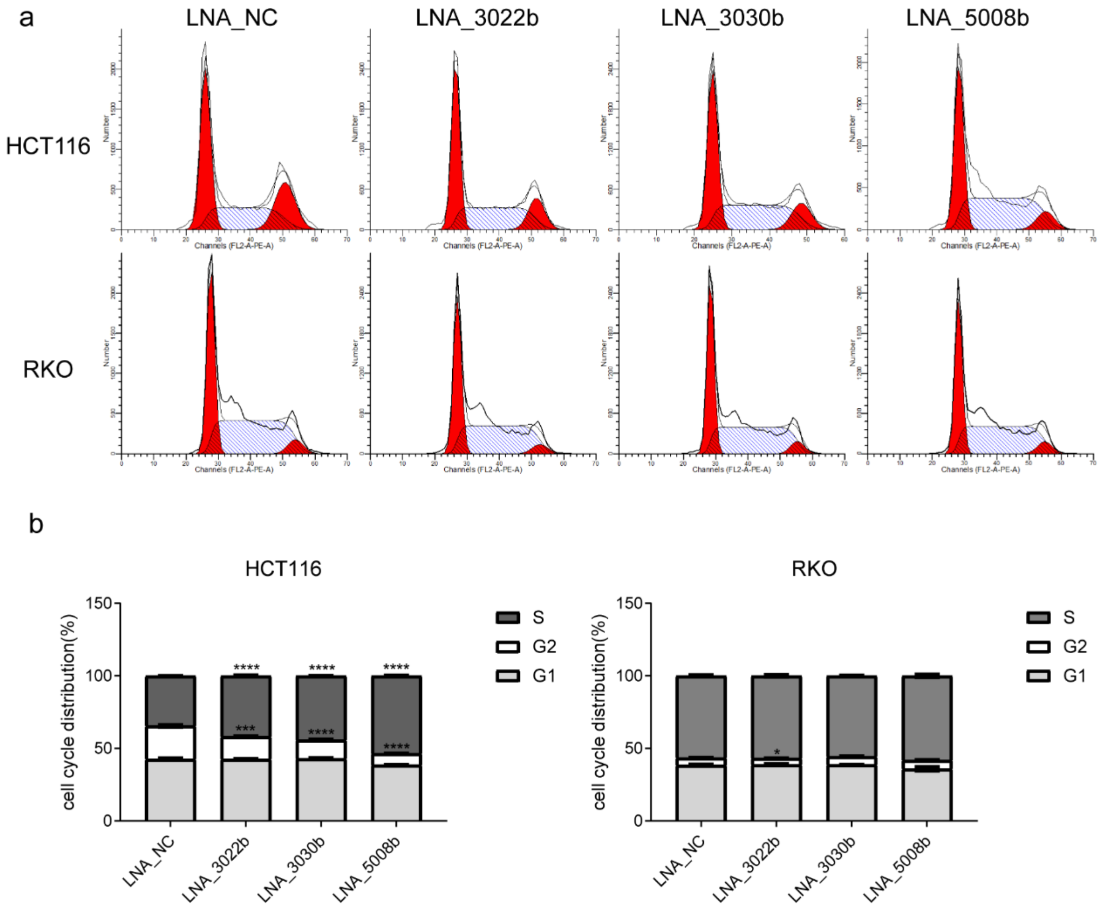

Figure S6. Knockdown of tRF-3022b, tRF-3030b, and tRF-5008b arrested the G2/M phases. (a) Cell cycle analysis of HCT116 and RKO cells transfected with LNA_3022b, LNA_3030b and LNA_5008b compared with LNA_NC using PI staining by flow cytometry. (b) Stacked bar plots show the percentages indicating cells in the G1 phase, S phase and G2 phase. (*$P < 0.05$, ****$P < 0.0001$).

Figure S7

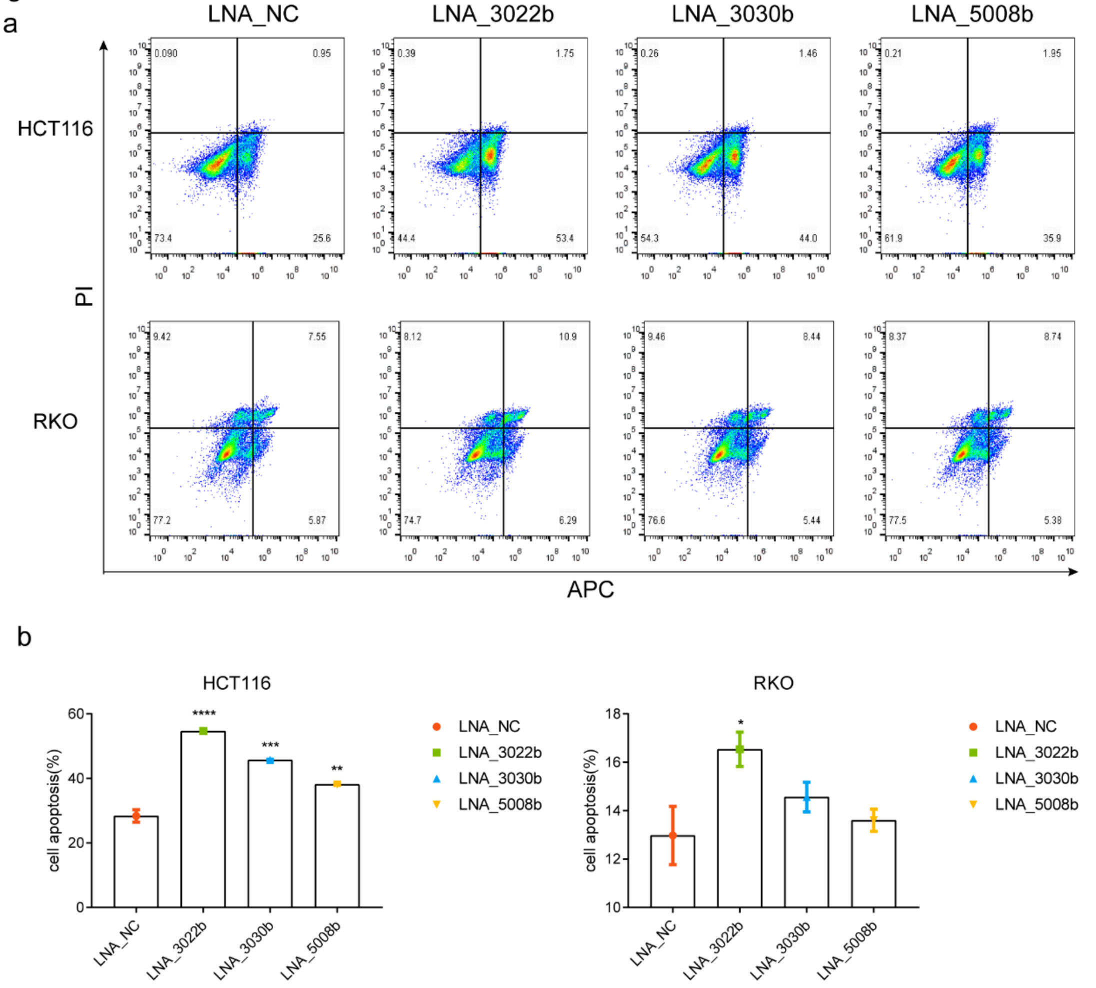

Figure S7. Knockdown of tRF-3022b promoted cellular apoptosis. (a) Apoptosis among tRF-3022b, tRF-3030b and tRF-5008b knockdown HCT116 and RKO cells and their negative controls were detected using flow cytometry. (b) Bar plots show the results of the quantitative analysis of apoptotic cells (%). (*$P < 0.05$, **$P < 0.01$, ***$P < 0.001$, ****$P < 0.0001$).

Figure S8

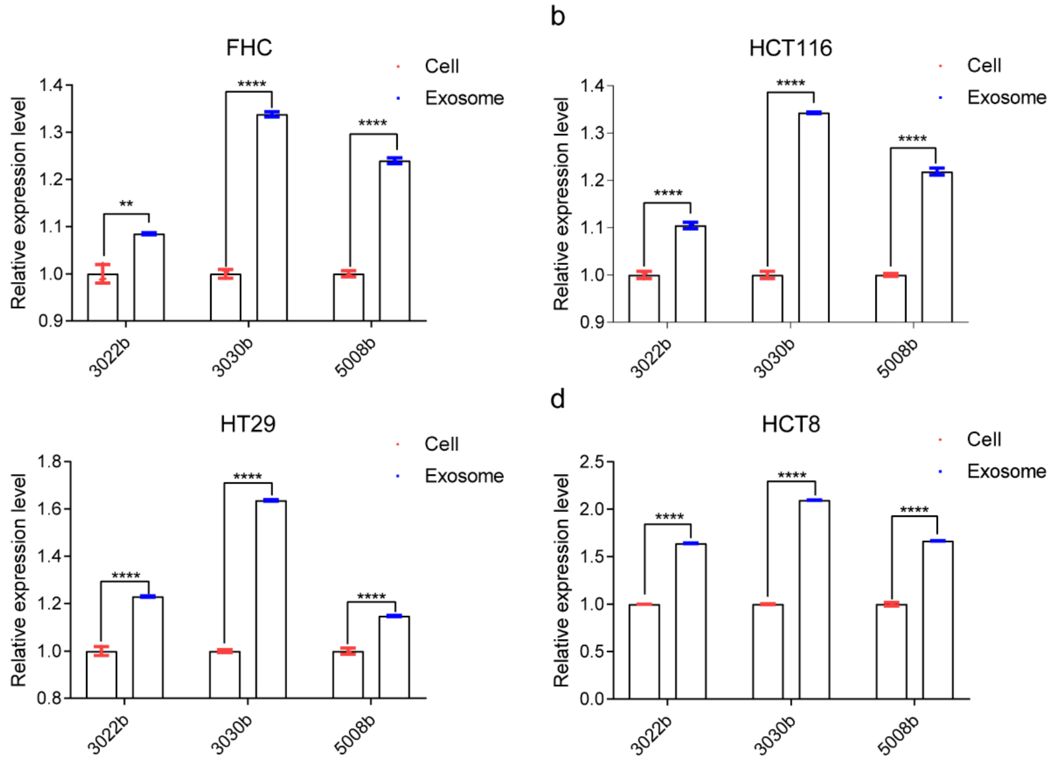

Figure S8. Quantification of tRF-3022b, tRF-3030b and tRF-5008b in both cells and exosomes. (a) FHC cells (b) HCT116 cells (c) HT29 cells (d) HCT8 cells. (**$P < 0.01$, ****$P < 0.0001$).

Figure S9

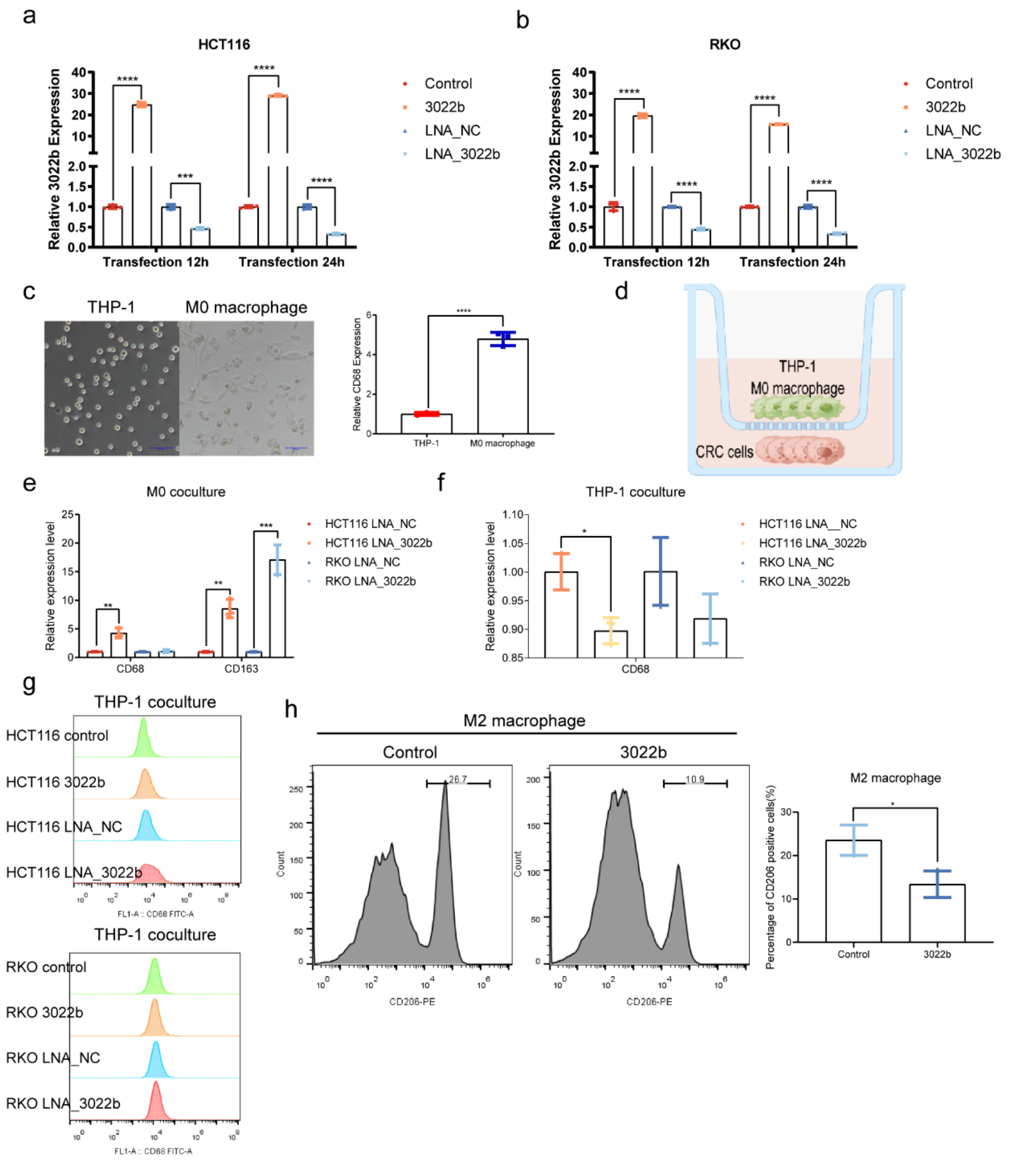

Figure S9. tRF-3022b regulates M2 macrophage polarization. (a-b) The efficiency of upregulated and knockdown tRF-3022b was detected by qRT-PCR. (c) Morphology of THP-1 and M0 macrophages (induced by PMA) and expression of CD68 as measured by qRT-PCR. (d) Schematic of co-culture assay of THP-1, M0 macrophages, and CRC cells. (e) qRT-PCR analysis of CD68 and CD163 in M0 macrophages co-cultured with tRF-3022b-knockdown or negative control HCT116 and RKO. (f) and (g) CD68 expression was detected by qRT-PCR and flow cytometric analysis in THP-1 co-cultured with tRF-3022b-knockdown or negative control HCT116 and RKO, respectively. (h) Flow cytometry was performed to quantify the expression of CD206 in tRF-3022b-knockdown M2 macrophages. (*$P < 0.05$, **$P < 0.01$, ***$P < 0.001$, ****$P < 0.0001$).

Figure S10

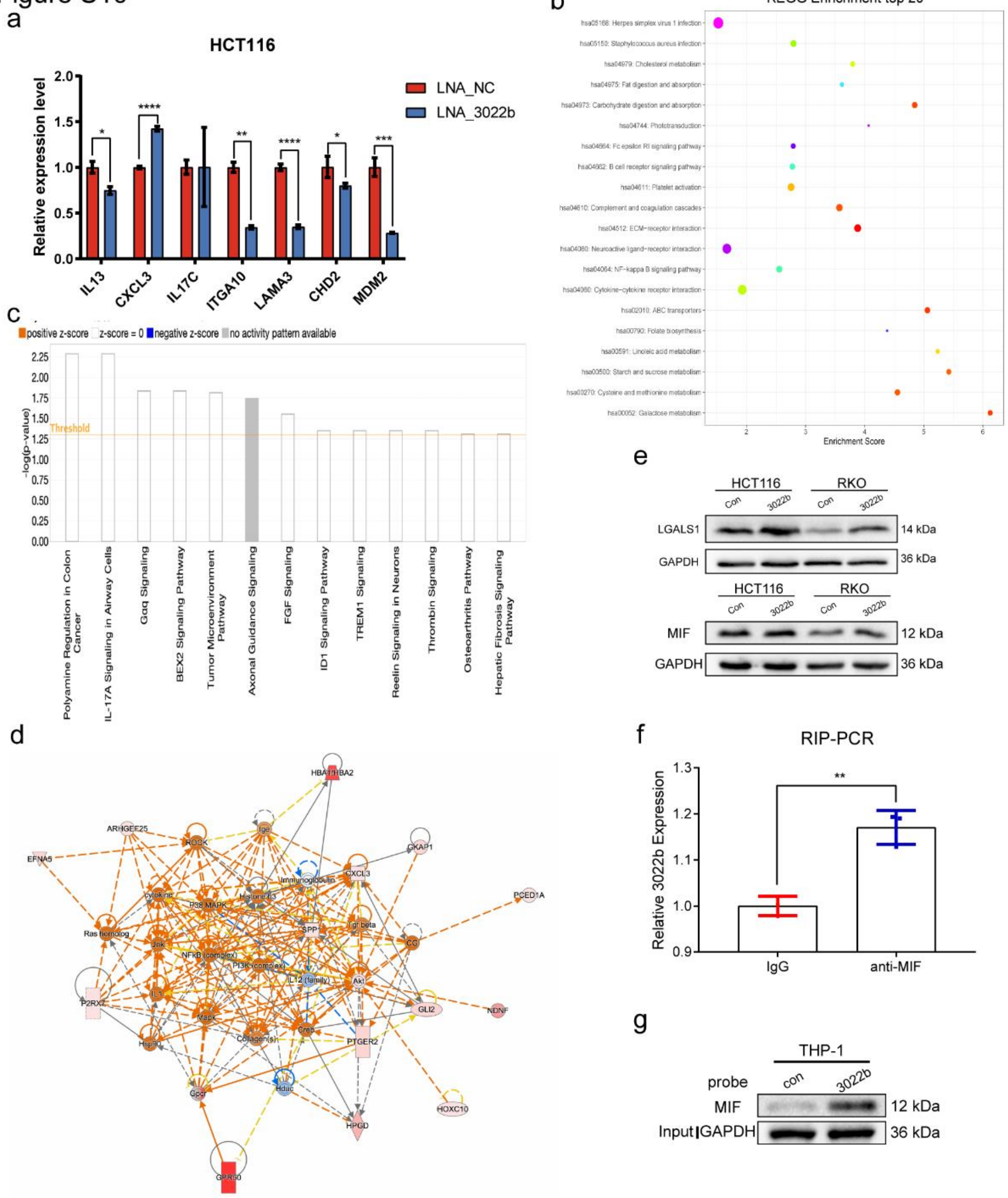

Figure S10. tRF-3022b contributes to the tumor microenvironment. (a) The mRNA expression levels of various DEGs in tRF-3022b-overexpressing HCT116 were measured using qRT-PCR. (b) Bubble heatmap of the top 20 abundant KEGG pathway annotations in tRF-3022b-overexpressing HCT116 versus negative control HCT116. (c) Pathway enrichment and (d) top network identified using Ingenuity pathway analysis (IPA) in tRF-3022b-overexpressing HCT116 versus negative control HCT116. (e) Protein levels of LGALS1 and MIF in CRC cells with tRF-3022b overexpression were detected by western blot, respectively. (f) Association between tRF-3022b and MIF was detected by the RIP assay in HCT116 cells. (g) Western blot analysis of MIF pulled down by tRF-3022b in THP-1 cells. (**P* < 0.05, ***P* < 0.01, ****P* < 0.001, *****P* < 0.0001).

Figure S11

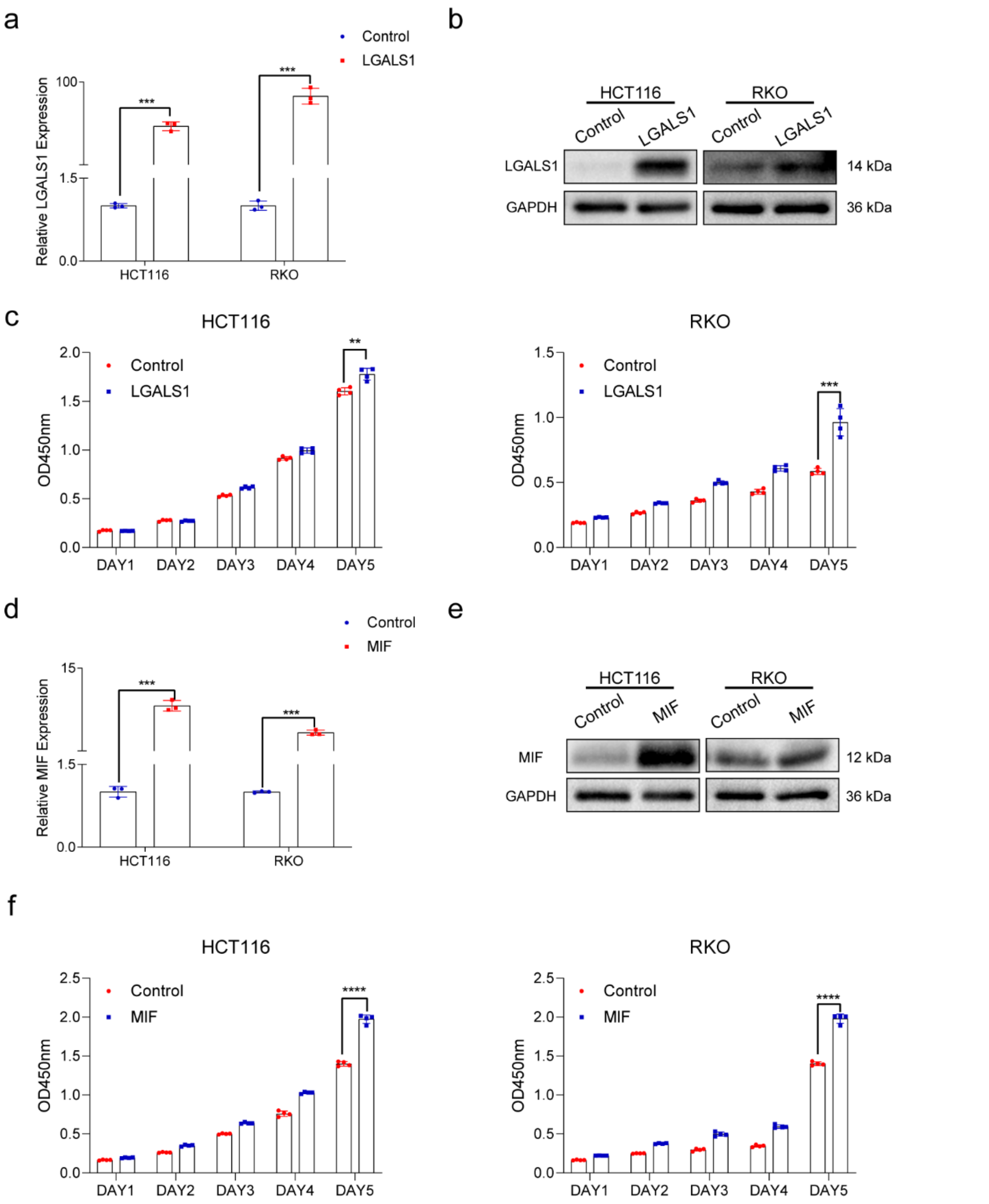

Figure S11. LGALS1 and MIF promote CRC cell proliferation *in vitro*. Transfection efficiency of LGALS1 (a-b) and MIF (d-e) was measured by qRT-PCR and western blot. (c, f) Cell proliferation assays for CRC cells. (***P* < 0.01, ****P* < 0.001, *****P* < 0.0001).
