# Supplementary material for: A novel tRNA-derived fragment tRF-3022b modulates cell apoptosis and M2 macrophage polarization via binding to cytokines in colorectal cancer
Source: J Hematol Oncol. 2022 Dec 16;15:176. doi: 10.1186/s13045-022-01388-z (PMC9756499; doi:10.1186/s13045-022-01388-z)
Supplement: Supplementary file 3 — Additional file 3. Table S1-1: Basic characteristics of patients who provided tissues and plasma exosomes for transcriptome sequencing and small RNA sequencing. Table S1-2: Basic characteristics of healthy controls who provided plasma exosomes for transcriptome sequencing and small RNA sequencing. Table S2: Clinical information of CRC patients who provided tissues for validation. Table S3: Clinical information of CRC patients who provided plasma exosomes for validation. Table S4: Primers and probes were used in this study. Table S5: Sequences of siRNAs and mimics or locked nucleic acids (LNA) were used in this study. Table S6: Antibodies were used in this study. [file 13045_2022_1388_MOESM3_ESM.docx]

Table S1-1. Basic characteristics of patients who provided tissues and plasma exosomes for transcriptome sequencing and small RNA sequencing.

| **No.** | **Gender** | | **Age** | **Cancer subtype** | **TNM stage** | | **transcriptome sequencing** | **small RNA sequencing** |
| --- | --- | --- | --- | --- | --- | --- | --- | --- |
| 1 | | Male | 67 | Sigmoid colon carcinoma | | ⅢB | Yes | Yes |
| 2 | | Male | 71 | Rectal carcinoma | | ⅢB | Yes | Yes |
| 3 | | Male | 67 | Colon carcinoma | | ⅢB | Yes | Yes |
| 4 | | Male | 69 | Colon carcinoma | | ⅢB | Yes | Yes |
| 5 | | Male | 63 | Colon carcinoma | | ⅢC | Yes | Yes |
| 6 | | Male | 60 | Rectal carcinoma | | ⅢB | Yes | Yes |
| 7 | | Male | 67 | Colon carcinoma | | ⅢB | Yes | Yes |
| 8 | | Male | 55 | Sigmoid colon carcinoma | | ⅣA | Yes | Yes |
| 9 | | Male | 73 | Colon carcinoma | | ⅢC | Yes | Yes |
| 10 | | Male | 76 | Rectal carcinoma | | ⅢB | Yes | Yes |

Table S1-2. Basic characteristics of healthy controls who provided plasma exosomes for small RNA sequencing.

| **No.** | **Gender** | **Age** | **transcriptome sequencing** | **small RNA sequencing** |
| --- | --- | --- | --- | --- |
| 1 | Male | 53 | NO | Yes |
| 2 | Male | 56 | NO | Yes |
| 3 | Male | 53 | NO | Yes |
| 4 | Male | 54 | NO | Yes |
| 5 | Male | 71 | NO | Yes |
| 6 | Male | 65 | NO | Yes |
| 7 | Male | 77 | NO | Yes |
| 8 | Male | 70 | NO | Yes |
| 9 | Male | 73 | NO | Yes |
| 10 | Male | 76 | NO | Yes |

Table S2. Clinical information of CRC patients who provided tissues for validation.

| Case No. | | Gender | Age | Cancer subtype | TNM stage |
| --- | --- | --- | --- | --- | --- |
| 1 | Male | | 79 | Rectal carcinoma | Ⅰ |
| 2 | Female | | 67 | Rectal carcinoma | Ⅰ |
| 3 | Female | | 79 | Colon carcinoma | Ⅰ |
| 4 | Male | | 68 | Sigmoid colon carcinoma | Ⅰ |
| 5 | Male | | 77 | Colon carcinoma | Ⅰ |
| 6 | Male | | 45 | Rectal carcinoma | Ⅰ |
| 7 | Female | | 59 | Colon carcinoma | Ⅰ |
| 8 | Female | | 65 | Rectal carcinoma | Ⅰ |
| 9 | Male | | 56 | Rectal carcinoma | Ⅰ |
| 10 | Female | | 85 | Rectal carcinoma | Ⅰ |
| 11 | Female | | 72 | Rectal carcinoma | Ⅰ |
| 12 | Female | | 58 | Rectal carcinoma | Ⅰ |
| 13 | Male | | 74 | Rectal carcinoma | Ⅰ |
| 14 | Male | | 43 | Colon carcinoma | Ⅰ |
| 15 | Male | | 62 | Colon carcinoma | Ⅰ |
| 16 | Female | | 41 | Rectal carcinoma | Ⅰ |
| 17 | Male | | 78 | Rectal carcinoma | ⅡA |
| 18 | Male | | 60 | Colon carcinoma | ⅡA |
| 19 | Male | | 74 | Colon carcinoma | ⅡA |
| 20 | Male | | 66 | Colon carcinoma | ⅡA |
| 21 | Female | | 53 | Rectal carcinoma | ⅡA |
| 22 | Male | | 53 | Colon carcinoma | ⅡA |
| 23 | Male | | 51 | Colon carcinoma | ⅡA |
| 24 | Female | | 74 | Rectal carcinoma | ⅡA |
| 25 | Male | | 69 | Colon carcinoma | ⅡA |
| 26 | Female | | 46 | Colon carcinoma | ⅡA |
| 27 | Male | | 57 | Rectal carcinoma | ⅡA |
| 28 | Male | | 65 | Colon carcinoma | ⅡA |
| 29 | Female | | 66 | Colon carcinoma | ⅡA |
| 30 | Female | | 57 | Colon carcinoma | ⅡA |
| 31 | Male | | 57 | Rectal carcinoma | ⅡA |
| 32 | Male | | 87 | Colon carcinoma | ⅡA |
| 33 | Male | | 78 | Rectal carcinoma | ⅡA |
| 34 | Male | | 65 | Colon carcinoma | ⅡA |
| 35 | Male | | 62 | Colon carcinoma | ⅢB |
| 36 | Male | | 49 | Rectal carcinoma | ⅢB |
| 37 | Male | | 76 | Colon carcinoma | ⅢB |
| 38 | Male | | 60 | Rectal carcinoma | ⅢB |
| 39 | Male | | 54 | Rectal carcinoma | ⅢB |
| 40 | Female | | 50 | Rectal carcinoma | ⅢC |
| 41 | Male | | 76 | Colon carcinoma | ⅢB |
| 42 | Female | | 68 | Colon carcinoma | ⅢB |
| 43 | Female | | 65 | Colon carcinoma | ⅢB |
| 44 | Female | | 83 | Colon carcinoma | ⅢC |
| 45 | Male | | 71 | Rectal carcinoma | ⅢC |
| 46 | Male | | 47 | Colon carcinoma | ⅢB |
| 47 | Female | | 53 | Rectal carcinoma | ⅢA |
| 48 | Male | | 57 | Colon carcinoma | ⅢB |
| 49 | Female | | 64 | Colon carcinoma | ⅢC |
| 50 | Male | | 76 | Rectal carcinoma | ⅢC |
| 51 | Male | | 73 | Rectal carcinoma | ⅢB |

Table S3. Clinical information of CRC patients who provided plasma exosomes for validation.

| Case No. | Gender | Age | Cancer subtype | TNM stage |
| --- | --- | --- | --- | --- |
| 1 | Male | 62 | Rectal carcinoma | ⅡA |
| 2 | Male | 65 | Rectal carcinoma | Ⅰ |
| 3 | Female | 57 | Colon carcinoma | ⅡA |
| 4 | Male | 55 | Colon carcinoma | ⅡA |
| 5 | Male | 67 | Rectal carcinoma | Ⅰ |
| 6 | Female | 77 | Colon carcinoma | ⅢB |
| 7 | Female | 54 | Colon carcinoma | ⅡA |
| 8 | Male | 61 | Colon carcinoma | ⅡA |
| 9 | Male | 64 | Sigmoid colon carcinoma | ⅡA |
| 10 | Male | 65 | Rectal carcinoma | ⅢB |
| 11 | Male | 65 | Rectal carcinoma | Ⅰ |
| 12 | Female | 57 | Sigmoid colon carcinoma | Ⅰ |
| 13 | Male | 70 | Colon carcinoma | ⅡA |
| 14 | Male | 68 | Colon carcinoma | ⅡA |
| 15 | Male | 71 | Colon carcinoma | ⅢB |
| 16 | Male | 74 | Colon carcinoma | ⅡA |
| 17 | Male | 65 | Rectal carcinoma | ⅡA |
| 18 | Male | 62 | Rectal carcinoma | ⅡA |
| 19 | Male | 71 | Rectal carcinoma | ⅡA |

Table S4. Primers and probes were used in this study.

| **qRT-PCR primers for tRFs** | **Sequence (5’-3’) Forward** |  |
| --- | --- | --- |
| tRF-3022b | UCGAUCCCCGUACGGGCCACCA |  |
| tRF-3030b | UCGAUUCCGGCUCGAAGGACCA |  |
| tRF-5008b | GCGUUGGUGGUAUAGUGGUGAGC |  |
| The reverse primers of all the small RNA were used the mRQ 3’ universal primer and U6 primers were provided by manufacturer. | | |
|  |  |  |
| **Gene symbol** | **Sequence (5’-3’) Forward** | **Reverse** |
| ACTIN | AAGTGTGACGTGGACATCCGC | CCGGACTCGTCATACTCCTGCT |
| LGALS1 | TCGCCAGCAACCTGAATCTC | GCACGAAGCTCTTAGCGTCA |
| MIF | CTGCACAGCATCGGCAAGAT | AGTTGATGTAGACCCTGTCCG |
| ALKBH3 | AGCCACGAGTGATTGACAGAG | ACAAACAGACCCTAGATACACCT |
| ANG | CTGGGCGTTTTGTTGTTGGTC | GGTTTGGCATCATAGTGCTGG |
| AGO2 | TCCACCTAGACCCGACTTTGG | GTGTTCCACGATTTCCCTGTT |
| DNMT2 | TGCCAAGACGATTGAAGGCAT | GCAGGGAGGGCTCATTAAAAT |
| PPARγ | ACCAAAGTGCAATCAAAGTGGA | ATGAGGGAGTTGGAAGGCTCT |
| CD68 | CCCACCTGCTTCTCTCATTC | CGAGAATGTCCACTGTGCT |
| CD163 | GCGGGAGAGTGGAAGTGAAAG | GTTACAAATCACAGAGACCGCT |
| CD206 | CAATTCCTGGCGATACCTCAG | GCACAACTCCGGTGACATCAA |
| IL10 | GACTTTAAGGGTTACCTGGGTTG | TCACATGCGCCTTGATGTCTG |
| CXCL3 | CGCCCAAACCGAAGTCATAG | GCTCCCCTTGTTCAGTATCTTTT |
| IL13 | CCTCATGGCGCTTTTGTTGAC | TCTGGTTCTGGGTGATGTTGA |
| TGF-β | CAATTCCTGGCGATACCTCAG | GCACAACTCCGGTGACATCAA |
| MMP9 | AGACCTGGGCAGATTCCAAAC | CGGCAAGTCTTCCGAGTAGT |
| IL17C | CCACACTGCTACTCGGCTG | CACACGGTATCTCCAGGGTGA |
| ITGA10 | AACATCACCCACGCCTATTCC | GTTGGTAGTCACCTAAGTGGC |
| LAMA3 | TGCTCAACTACCGTTCTGCC | TCCAGTTCTTTTGCGCTTTGT |
| CHD2 | AGTCAGTCGGAAAGTGAGCAG | ACATCAGCTATCCGTTCCTTCT |
| MDM2 | GAATCATCGGACTCAGGTACATC | TCTGTCTCACTAATTGCTCTCCT |
|  |  |  |
| **RNA pull down probes (3’-biotin)** | **Sequence (5’-3’)** | |
| Control probe | CGACUUAAGCGAGCUAGUAACUG | |
| tRF-3022b probe | UCGAUCCCCGUACGGGCCACCA | |
|  |  | |
| **FISH probe (3’-FAM)** | **Sequence (5’-3’)** | |
| tRF-3022b probe | TGGTGGCCCGTACGGGGATCGA | |

Table S5. Sequences of siRNAs and mimics or locked nucleic acids (LNA) were used in this study.

| **siRNAs** | **Sequence (5’-3’)** |
| --- | --- |
| siALKBH3#1 | CAGAGAGGAUAUAACUUAUTT |
| siALKBH3#2 | CCGCAUUGAAGAGAACACUTT |
| siALKBH3#3 | CCUGUGCUGCGCACACUAATT |
| siDNMT2#1 | CAAAUUCAAGGCUACGAUATT |
| siDNMT2#2 | CGUGCAUGUAGUAGCUAAATT |
| siDNMT2#3 | GGACAGGGUCUGUGUUACATT |
| siAGO2#1 | GGUGAUAAAUACAGUACAATT |
| siAGO2#2 | GGAAAUAUGGUUUGCUAAATT |
| siAGO2#3 | GGAAUCUUGUGACUAAUAATT |
| siANG#1 | GGUCACCACUUGCAAGCUATT |
| siANG#2 | CCAGCACUAUGAUGCCAAATT |
| siANG#3 | GCAACAAGCGCAGCAUCAATT |
| Negative control | TTCTCCGAACGTGTCACGT |
|  |  |
| **Mimics** | **Sequence (5’-3’)** |
| control | CGACUUAAGCGAGCUAGUAACUG |
| tRF-3022b | UCGAUCCCCGUACGGGCCACCA |
|  |  |
| **LNA** | **Sequence (5’-3’)** |
| LNA_NC | CAGCGTAGCGGATACTCGAGTTC |
| LNA_3022b | TGGTGGCCCGTACGGGGATCGA |
| LNA_3030b | TGGTCCTTCGAGCCGGAATCGA |
| LNA_5008b | GCTCACCACTATACCACCAACGC |

Table S6. Antibodies were used in this study.

| Protein name | Applications | Product number |
| --- | --- | --- |
| TSG101 | WB | Proteintech, 14497-1-AP |
| CD9 | WB | Proteintech, 20597-1-AP |
| E-cadherin | WB | Proteintech, 20874-1-AP |
| LGALS1 | WB | ABclonal, A18040 |
| MIF | RIP | Abcam, ab175189 |
| Normal Rabbit IgG | RIP | CST, #2729S |
| LGALS1 | IF | CST, #12936 |
| MIF | IF, WB | CST, #75038 |
| CD68 | Flow | eBioscience, 12-2069-42 |
| CD206 | Flow | eBioscience, 11-0689-41 |
| ALKBH3 | WB | Proteintech, 12292-1-AP |
| DNMT2 | WB | Proteintech, 19221-1-AP |
| AGO2 | WB | Proteintech, 67934-1-Ig |
| ANG | WB | Proteintech, 18302-1-AP |
| GAPDH | WB | ABclonal, AC033 |
