# Supplementary material for: A novel tRNA-derived fragment tRF-3022b modulates cell apoptosis and M2 macrophage polarization via binding to cytokines in colorectal cancer
Source: J Hematol Oncol. 2022 Dec 16;15:176. doi: 10.1186/s13045-022-01388-z (PMC9756499; doi:10.1186/s13045-022-01388-z)
Supplement: Supplementary file 5 — Additional file 5. Table S21-1: Grouping of high tRF_group and low tRF_group in tissues. Table S21-2: Grouping of high tRF_group and low tRF_group in plasma exosomes. [file 13045_2022_1388_MOESM5_ESM.docx]

Table S21-1. Grouping of high tRF_group and low tRF_group in tissues.

| **high tRF_group** | **N8(0.2660063)** | **T6(0.2944841)** | **T7(0.4372818)** | **T10(0.4925912)** | **T2(0.6903274)** |
| --- | --- | --- | --- | --- | --- |
| **low tRF_group** | **N4(-0.30967264)** | **N2(-0.26829155)** | **N3(-0.22387942)** | **N6(-0.07272661)** | **T4(0.08579416)** |

The scores of tRFs in patients providing tissues

| **Normal tissue** | **N10** | **N2** | **N3** | **N4** | **N5** | **N6** | **N7** | **N8** | **N9** |
| --- | --- | --- | --- | --- | --- | --- | --- | --- | --- |
| **scores** | 0.194172 | -0.26829 | -0.22388 | -0.30967 | 0.143923 | -0.07273 | 0.194312 | 0.266006 | 0.139746 |
| **Tumor tissue** | **T10** | **T2** | **T3** | **T4** | **T5** | **T6** | **T7** | **T8** | **T9** |
| **scores** | 0.492591 | 0.690327 | 0.245454 | 0.085794 | 0.233396 | 0.294484 | 0.437282 | 0.153046 | 0.09904 |

Table S21-2. Grouping of high tRF_group and low tRF_group in plasma exosomes.

| **high tRF_group** | **T6(0.1307816)** | **T3(0.1641662)** | **T7(0.2492084)** | **T10(0.3029463)** | **T2(0.5657125)** |
| --- | --- | --- | --- | --- | --- |
| **low tRF_group** | **N4(-0.4342875)** | **N2(-0.3349534)** | **N6(-0.3220115)** | **N3(-0.2389817)** | **N5(-0.2091254)** |

The scores of tRFs in patients and healthy controls providing plasma exosomes

| **Healthy controls** | **N10** | **N2** | **N3** | **N4** | **N5** | **N6** | **N7** | **N8** | **N9** |
| --- | --- | --- | --- | --- | --- | --- | --- | --- | --- |
| **scores** | 0.118284785001629 | -0.33495339960648 | -0.238981724100929 | -0.434287471336704 | -0.209125409533274 | -0.322011463679312 | 0.0900511161666768 | 0.0731420257273104 | 0.114719976038013 |
| **CRC patients** | **T10** | **T2** | **T3** | **T4** | **T5** | **T6** | **T7** | **T8** | **T9** |
| **scores** | 0.302946349901911 | 0.565712528663296 | 0.164166190501227 | -0.0375184797724253 | 0.0864244022616432 | 0.130781587915083 | 0.249208416692876 | -0.0202499375680219 | -0.0360715068859815 |
